# Linking diabetic retinopathy with lipid abnormalities

**DOI:** 10.6026/973206300220128

**Published:** 2026-01-31

**Authors:** Yasha Bandil, Pankaj Kumar Gupta, Rakesh Manglani, Rahul Soni, Sachin Parmar

**Affiliations:** 1 Department of Ophthalmology, BirsaMunda Government Medical College Shahdol, Madhya Pradesh, India; 2 Department of General Medicine, BirsaMunda Government Medical College Shahdol, Madhya Pradesh, India; 3 Department of Biochemistry, BirsaMunda Government Medical College, Shahdol, Madhya Pradesh, India; 4 Department of Pulmonary Medicine, BirsaMunda Government Medical College Shahdol, Madhya Pradesh, India; 5 Department of Community Medicine, V.K.S. Government Medical College, Neemuch, Madhya Pradesh, India

**Keywords:** Diabetic retinopathy, dyslipidemia, Type 2 diabetes mellitus

## Abstract

Diabetic retinopathy (DR) is a leading cause of preventable blindness in diabetes mellitus. Therefore, it is of interest to investigate the association between dyslipidemia and DR in 100 patients, divided into 50 with DR and 50 without DR. Lipid Abnormalities was found in 57% of participants, with a higher prevalence of DR in those with Lipid Abnormalities (35/57). The study also identified that the duration of diabetes significantly influenced DR development. Thus, a strong association between Lipid Abnormalities and DR, warranting further research on lipid-lowering interventions to prevent DR.

## Background:

Diabetes mellitus is regarded as a disease that affects all organs. A significant number of individuals with type 2 diabetes exhibit macrovascular and microvascular complications at the initial diagnosis of the condition [1, 2-3]. Diabetic retinopathy (DR) is a vascular condition that impacts the microvasculature of the retina. Diabetes mellitus is estimated to affect 4 percent of the global population, with nearly half exhibiting some degree of diabetic retinopathy at any given moment. Epidemiological data from the past decade indicate that the pattern and profile of type 2 diabetes mellitus in India significantly differ from those in the West [4]. A prior study conducted on a clinic-based population of diabetes mellitus patients indicated an overall prevalence of 14 percent. NPDR was noted in 6 percent, whereas 4 percent exhibited macular edema and 4 percent presented with PDR [5]. The Asian Young Diabetes Research (ASDIAB) Study reported the incidence of diabetic retinopathy (DR) in 724 young diabetic individuals aged 12 to 40 years, with a diabetes duration of less than 12 months, across seven centers in four Asian countries. Notably, the prevalence of diabetic retinopathy (DR) was lowest among Indians at 5.3%, in contrast to other ethnic groups such as Malays at 10% and Chinese at 15.1% [6]. Initial data from the hospital indicates a predominance of non-obese type 2 diabetes mellitus, accompanied by a high incidence of complications. In light of the aforementioned observations, the current study was formulated to examine lipid levels in patients with diabetes mellitus and subsequently analyze the data concerning the incidence of diabetic retinopathy. Therefore, it is of interest to describe the linking of diabetic retinopathy with lipid abnormalities.

## Methods:

This was an observational case-control study conducted among patients attending a diabetic clinic. A total of 100 patients who met the inclusion criteria were enrolled in the study. These patients were equally divided into two groups: 50 cases, consisting of diabetics with retinopathy (DR), and 50 controls, consisting of diabetics without retinopathy (No DR). The primary objective was to analyze the association between diabetic retinopathy and Lipid Abnormalities. The diagnosis of diabetes mellitus was made based on the criteria set forth by the American Diabetes Association (ADA). Classification of patients into the DR and No DR groups was based on optic fundus examination findings. Patients diagnosed with diabetic retinopathy were further categorized into those with non-proliferative diabetic retinopathy (NPDR) and proliferative diabetic retinopathy (PDR). Lipid Abnormalities was defined according to the National Cholesterol Education Program Adult Treatment Panel III (NCEP ATP III) guidelines. Body Mass Index (BMI) was calculated as weight in kilograms divided by the square of height in meters (kg/m^2^) and BMI categorization was done in accordance with Indian guidelines. Patients who had associated hypertension or were taking medications known to influence lipid or lipoprotein metabolism-such as diuretics, beta-blockers, hypolipidemic agents, or hormonal treatments-were excluded from the study to eliminate confounding factors. All patients underwent relevant investigations including Fasting Blood Sugar (FBS), Postprandial Blood Sugar (PPBS), HbA1c, complete blood count, and fasting lipid profile.

## Results:

Table 1 presents the BMI distribution among study groups. Both the case and control groups consisted of 100 individuals each. In both groups, 10% of patients had a BMI of less than 18.5, while 23% fell within the 18.5-22.9 range. The majority, 53%, had a BMI between 23-27.9 and 14% had a BMI of 28 or above. This indicates a similar distribution of BMI across both cases and controls. Table 2 shows the distribution of Lipid Abnormalities among patients with and without diabetic retinopathy (DR). Out of 100 patients, 57 had Lipid Abnormalities, among whom 35 had DR and 22 did not. In contrast, among the 43 patients without Lipid Abnormalities, 15 had DR and 28 did not. These findings suggest a higher occurrence of diabetic retinopathy among patients with Lipid Abnormalities. Table 3 outlines the gender distribution of the study population. Of the 100 patients included, 57 were male and 43 were female, making up 57% and 43% of the total population, respectively. This indicates a slight male predominance in the sample. Table 4 illustrates the age-wise distribution of patients. The most common age group was 50-59 years, comprising 37% of the patients. This was followed by 27% in the 40-49 years group, 23% in the 60-69 years group, and 13% in the 70-79 years group. This suggests that the majority of participants were middle-aged. Table 5 describes the duration of diabetes among the study population. Thirty percent of patients had diabetes for less than 5 years, 36% had it for 5-10 years and 34% had been living with diabetes for over 10 years. This reflects a fairly even distribution across the three categories, with a slight predominance in the 5-10 year range.

## Discussion:

The findings from this study highlight significant associations between lipid abnormalities, diabetes mellitus (DM), and diabetic retinopathy (DR), consistent with existing literature. Below, we discuss each result in the context of prior research. Both cases and controls showed similar BMI distributions, with 53% in the 23-27.9 kg/m^2^ range. Elevated BMI is a well-established risk factor for DM and its complications. Studies indicate that higher BMI correlates with increased prevalence of Lipid Abnormalities, hypertension, and DR due to insulin resistance and chronic inflammation [7, 8]. For instance, individuals with BMI ≥ 30 kg/m^2^ exhibit a 2-3 times higher risk of DR progression compared to those with normal BMI [7, 9]. This aligns with our cohort, where overweight/obese individuals (BMI ≥ 23 kg/m^2^) constituted 67% of the sample, potentially exacerbating metabolic dysregulation. A strong association was observed between Lipid Abnormalities and DR, with 61.4% of DR patients having Lipid Abnormalities versus 44% without DR. This aligns with studies demonstrating that Lipid Abnormalities-particularly low HDL and elevated triglycerides-accelerates retinal microvascular damage [10, 11]. For example, a study by Khan et al. found that DR patients had significantly lower HDL (38.5 mg/dL vs. 47.1 mg/dL) and higher triglycerides (179.9 mg/dL vs. 160.6 mg/dL) [10]. Similarly, oxidized LDL and lipid peroxidation contribute to blood- retinal barrier breakdown, worsening DR severity [12]. Our findings reinforce the need for aggressive lipid management in DM patients to mitigate retinopathy risk. Males constituted 57% of the cohort, contrasting with epidemiological data showing higher DM prevalence in men but greater complication risks in women [12, 13]. For instance, women with DM face a 27% higher cardiovascular risk than men [12], yet our male predominance may reflect regional screening practices or sampling bias. Further studies should explore gender-specific pathways in DR progression. The majority of patients were aged 50-59 years (37%), consistent with CDC data showing diabetes prevalence peaks in older adults (29.2% in ≥65 years) [13]. Longer DM duration (>10 years) was observed in 34% of patients, correlating with DR severity. This mirrors findings where DR prevalence rises from 12% at 5-10 years to 58.7% after 25-30 years [15]. A nonlinear relationship exists, with DR risk escalating sharply within the first 8 years of DM diagnosis before plateauing [16]. Early glycemic control is critical, as prolonged hyperglycemia induces retinal oxidative stress and vascular dysfunction [17, 18].

## Conclusion:

The interplay of dyslipidemia, BMI, and DM duration in DR pathogenesis is shown. Limitations include a modest sample size and single- center design. Future research should validate these findings in larger, multiethnic cohorts and explore targeted lipid-lowering therapies to reduce DR burden.

## Figures and Tables

**Table 1:** BMI in study groups

**BMI Range**	**Case**	**Control**
<18.5	10	10
18.5-22.9	23	23
23-27.9	53	53
≥28	14	14
Total	100	100

**Table 2:** Lipid abnormalities in cases and controls

**Group**	**DR**	**No DR**	**Total**
Lipid Abnormalities	35	22	57
No Lipid Abnormalities	15	28	43
Total	50	50	100

**Table 3:** Gender distribution

**Gender**	**Frequency**	**Percentage**
Male	57	57%
Female	43	43%
Total	100	100%

**Table 4:** Age group distribution

**Age Group**	**Frequency**	**Approx. %**
40-49 years	27	27%
50-59 years	37	37%
60-69 years	23	23%
70-79 years	13	13%
Total	100	100%

**Table 5:** Duration of diabetes

**Duration of Diabetes**	**Frequency**	**Percentage**
<5 years	30	30%
5-10 years	36	36%
>10 years	34	34%
Total	100	100%
